# Incidental Finding of Giant Coronary Artery Aneurysms Successfully Treated with Medical Therapy

**DOI:** 10.1155/2019/7185383

**Published:** 2019-05-08

**Authors:** Rony Shammas, Prasanna Sengodan, Assad Movahed

**Affiliations:** Division of Cardiology, Vidant Medical Center-East Carolina University at Brody School of Medicine, Greenville, NC, USA

## Abstract

We report a case of a 30-year-old male who presented with signs and symptoms of respiratory infection with left lower lobe consolidation and cardiomegaly on a chest radiography. The presence of cardiomegaly lead to further cardiac evaluation revealing giant coronary aneurysms. The patient was treated conservatively with Coumadin and aspirin and has done well at four years of follow-up.

## 1. Background

Coronary artery aneurysms (CAAs) are defined as a focal dilation of coronary segments of at least 1.5 times the adjacent normal segment, whereas the term coronary artery ectasia is used to define similar, but more diffuse, lesions. The overall incidence ranges from 0.3 to nearly 5%. With more widespread use of coronary angiography, CAAs have been increasingly identified as an incidental finding. Giant CAAs defined as dilation of the artery greater than 4 times the reference diameter are rare.

## 2. Case Description

A 30-year-old male with a history of cerebral palsy, autism, and scoliosis presented to the emergency department with complaints of left-sided pleuritic chest pain, shortness of breath, and fever.

**Figure 1:**
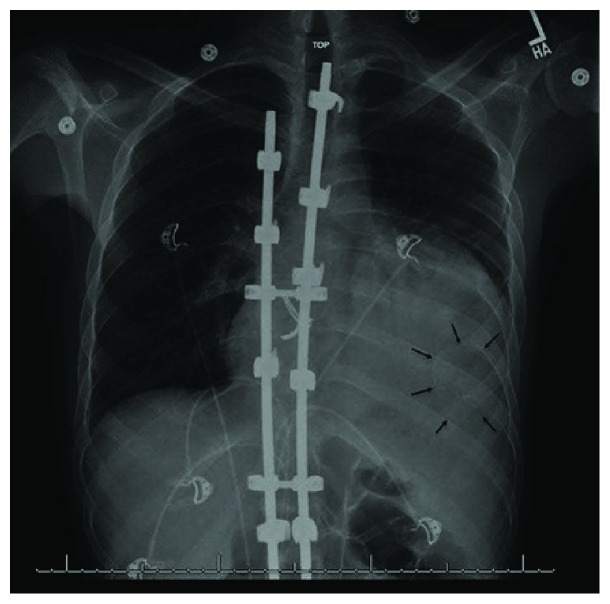
Chest radiography showing left lower lobe consolidation, cardiomegaly, and a calcified mass in the left lung base.

**Figure 2:**
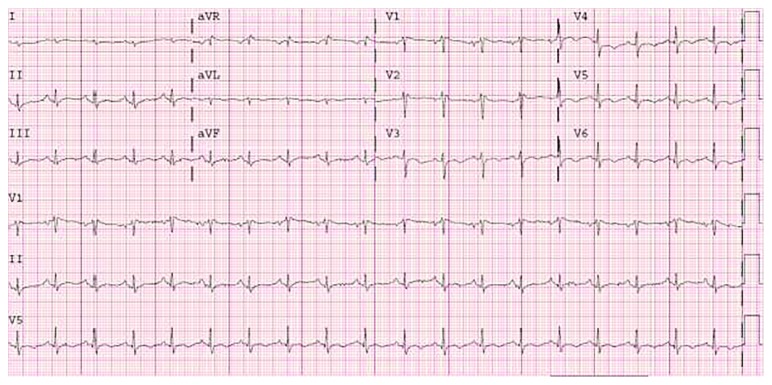
Electrocardiogram (EKG) showed sinus tachycardia with a rate of 114 with right axis deviation and nonspecific ST-T changes.

**Figure 3:**
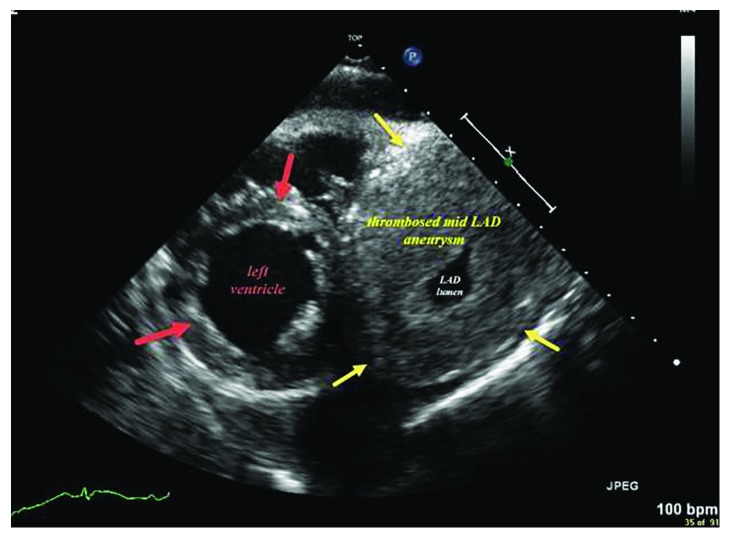
Two-dimensional transthoracic echocardiogram short axis view showing a large extracardiac mass (yellow arrows) alongside the left ventricle (red arrows).

Initial work-up revealed a white blood cell count of 19,300 and a chest radiography (Figure 1) showed left lower lobe consolidation, cardiomegaly, and a calcified mass in the left lung base. Electrocardiogram (EKG) showed sinus tachycardia with a rate of 114 with right axis deviation and non-specific ST-T changes (Figure 2). He was initially treated for pneumonia and subsequently underwent an echocardiogram (Figure 3) due to findings of cardiomegaly on the chest radiograph. This revealed a large extra cardiac mass alongside the left ventricle with normal left and right ventricular size and function.

**Figure 4:**
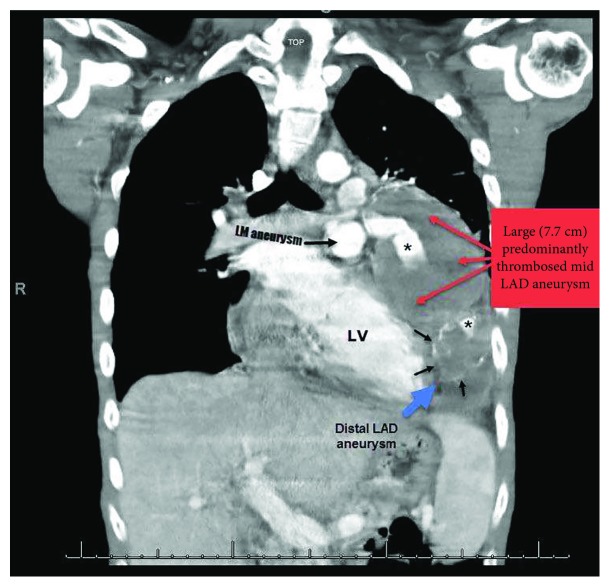
Computed tomographic scan shows a large mostly thrombosed proximal and mid left anterior descending artery (LAD) aneurysm (red arrows) measuring 7.7 cm in addition to a smaller calcified distal aneurysm (blue arrow). The opacified left main (LM) aneurysm is also seen.

**Figure 5:**
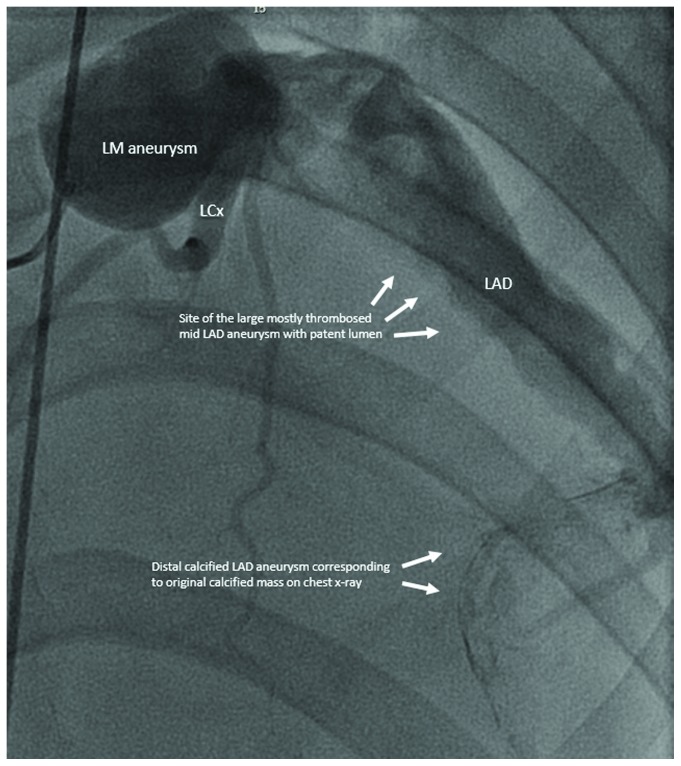
Coronary angiography showing multiple aneurysms within the left main (LM) coronary artery and left anterior descending artery (LAD) with slow flow. The top arrows point to the large mostly thrombosed aneurysm and the lower arrows show the location of the smaller calcified distal aneurysm.

Computed tomographic scan of the chest (Figure 4) showed large mostly thrombosed proximal and mid left anterior descending artery (LAD) aneurysm measuring 7.7 cm in addition to a smaller calcified distal aneurysm which corresponds to the mass seen on the chest radiography. The LAD lumen appeared to be patent (asterisk). Coronary angiography (Figure 5) confirmed the presence of multiple aneurysms within the left main coronary artery and LAD with slow flow. The large mid LAD aneurysm was not well delineated on the angiogram due to the absence of calcification. No aneurysmal changes were noted in the right coronary or left circumflex arteries.

Due to the extensive and diffuse nature of the aneurysmal changes which involved the whole length of the LAD in addition to the substantial amount of organized thrombus, surgical intervention was not felt to be feasible. The patient was placed on Coumadin, atorvastatin, and aspirin and has not had a cardiac event over a 4-year follow-up period.

## 3. Discussion

Coronary artery aneurysms are defined as a focal dilation of coronary segments of at least 1.5 times the adjacent normal segment, whereas the term coronary artery ectasia is used to define similar, but more diffuse, lesions [1]. The right coronary artery is usually the most affected artery (40%) followed by the left anterior descending (32%), and the left main being the least affected artery (3.5%) [2]. Mechanisms underlying their formation including a molecular basis (a possible role of matrix metalloproteinases (MMPs)) are being investigated as suggested by Lamblin et al. [3]. They report that the 5A/5A genotype of MMP-3 was significantly more frequent in patients with coronary aneurysms than in controls. The pathogenesis of the formation of CAAs is not clear; however, a few mechanisms have been proposed including individual genetic susceptibility, atherosclerosis, and iatrogenic injury following percutaneous interventions, in addition to congenital etiology, vasculitis, and connective tissue disorders such as Kawasaki disease and Marfan syndrome. Fibromuscular dysplasia, a nonatherosclerotic and non-inflammatory vascular disease, commonly associated with lesions of the internal carotid and renal arteries, has also been linked to CAA [4].

The two common classifications of CAAs are either saccular or fusiform. Saccular aneurysms are found to be more common in the left anterior descending artery than in other coronary arteries [5, 6]. The clinical presentation of CAAs can vary widely and mostly depends on the underlying etiology. The slow flow of blood on the irregular internal surface of the aneurysm wall predisposes to the formation of thrombi with potential for subsequent embolization, resulting in ischemic symptoms [6–9].

Although CAAs are mostly detected incidentally during angiography, they are much less likely to show up on a chest radiograph or echocardiogram as in our case. Other useful modalities for diagnosis are computed tomography and cardiac magnetic resonance angiography. During angiography, delayed antegrade contrast filling, segmental back flow, and contrast stasis in the dilated coronary segment often hamper optimal imaging [10]. Intravascular ultrasound can be extremely helpful to supplement angiography and may be considered to help distinguish between true aneurysm, pseudoaneurysm, and segments with aneurysmal appearance which may be due to stenosis.

Since the natural history and prognosis are related to multiple factors, the decisions around treatment should be tailored to each patient and should consider many aspects such as the clinical presentation, etiology, aneurysm size, location, association with infections, and the presence and extent of any coexisting atherosclerosis [2, 11, 12]. In general, the smaller the size of the aneurysm and earlier the treatment is initiated, the lower the chance of major adverse cardiac outcomes [13–15]. Doi et al. suggested a possible advantage of anticoagulation in patients with CAA and acute coronary syndrome [16]. Percutaneous intervention may be performed in certain cases, using covered stents; however, substantial thrombus burden, sizing, and landing zone assessment may be problematic. Surgical options include resection of the aneurysm, proximal and/or distal ligation, and aneurysmal thrombectomy, with or without bypass grafting. The role of newer anticoagulants in the management of CAAs remains to be studied.
